# Influence of Surface Ligand Density and Particle Size on the Penetration of the Blood–Brain Barrier by Porous Silicon Nanoparticles

**DOI:** 10.3390/pharmaceutics15092271

**Published:** 2023-09-03

**Authors:** Weisen Zhang, Douer Zhu, Ziqiu Tong, Bo Peng, Xuan Cheng, Lars Esser, Nicolas H. Voelcker

**Affiliations:** 1 Drug Delivery, Disposition and Dynamics, Monash Institute of Pharmaceutical Sciences, Monash University, Parkville, VIC 3052, Australiatommy.tong@monash.edu (Z.T.);; 2 Frontiers Science Center for Flexible Electronics, Xi’an Institute of Flexible Electronics (IFE), Xi’an Institute of Biomedical Materials & Engineering (IBME), Northwestern Polytechnical University, Xi’an 710072, China; 3 Commonwealth Scientific and Industrial Research Organisation (CSIRO), Clayton, VIC 3168, Australia; heidi.cheng@csiro.au; 4 Melbourne Centre for Nanofabrication, Victorian Node of the Australian National Fabrication Facility, Clayton, VIC 3168, Australia; 5 Department of Materials Science and Engineering, Monash University, Clayton, VIC 3800, Australia

**Keywords:** blood–brain barrier, nanoparticles, porous silicon nanoparticles, BBB-on-a-chip, ligand density, organ-on-a-chip, nanomedicine, microfluidic model

## Abstract

Overcoming the blood–brain barrier (BBB) remains a significant challenge with regard to drug delivery to the brain. By incorporating targeting ligands, and by carefully adjusting particle sizes, nanocarriers can be customized to improve drug delivery. Among these targeting ligands, transferrin stands out due to the high expression level of its receptor (i.e., transferrin receptor) on the BBB. Porous silicon nanoparticles (pSiNPs) are a promising drug nanocarrier to the brain due to their biodegradability, biocompatibility, and exceptional drug-loading capacity. However, an in-depth understanding of the optimal nanoparticle size and transferrin surface density, in order to maximize BBB penetration, is still lacking. To address this gap, a diverse library of pSiNPs was synthesized using bifunctional poly(ethylene glycol) linkers with methoxy or/and carboxyl terminal groups. These variations allowed us to explore different transferrin surface densities in addition to particle sizes. The effects of these parameters on the cellular association, uptake, and transcytosis in immortalized human brain microvascular endothelial cells (hCMEC/D3) were investigated using multiple in vitro systems of increasing degrees of complexity. These systems included the following: a 2D cell culture, a static Transwell model, and a dynamic BBB-on-a-chip model. Our results revealed the significant impact of both the ligand surface density and size of pSiNPs on their ability to penetrate the BBB, wherein intermediate-level transferrin densities and smaller pSiNPs exhibited the highest BBB transportation efficiency in vitro. Moreover, notable discrepancies emerged between the tested in vitro assays, further emphasizing the necessity of using more physiologically relevant assays, such as a microfluidic BBB-on-a-chip model, for nanocarrier testing and evaluation.

## 1. Introduction

Neurological disorders are a global health challenge and the second leading cause of mortality, with more than 10 million deaths reported globally in 2019 alone [1]. Diseases associated with neurological disorders, such as Alzheimer’s disease, Parkinson’s disease, and brain cancers currently affect over 349 million lives, sadly, with no effective treatments on hand [1,2,3]. Although pre-clinical research has resulted in the discovery of multiple promising drug candidates, clinical translation has trailed behind, as the presence of the blood–brain barrier (BBB) prevents the delivery of most drugs to the brain [4,5,6]. The BBB is a highly efficient physical barrier that consists of endothelial cells and other cells, such as pericytes and astrocytes [7]. The BBB functions as a vascular hurdle between the blood and the brain, blocking most blood constituents from entering the brain, as the BBB’s tight junctions prevent paracellular transport; moreover, pinocytic activity is also very restricted [8,9].

Several strategies, such as focused ultrasounds [10], implantable reservoirs [11], and convection-enhanced injections [12], have been reported to help therapeutic molecules cross the BBB. Nevertheless, these technologies possess several limitations. For example, focused ultrasounds display limitations with regard to the reproducibility of clinical procedures, and brain implants are associated with infection risks due to invasive surgical procedures and the implant materials used [13,14]. On the other hand, nanoparticles are a promising technology as they promote BBB penetration, and they target diseased regions in the brain while limiting side effects [15,16,17,18]. For example, so-called “sequential targeting interlocking” nanoparticles were shown to cross the BBB and precisely target brain cancer [19]. This system was developed using a ligand for the glucose receptor, GLUT1, to promote BBB transcytosis, and it contained a pH-responsive linker to promote drug payload release in the acidic tumor microenvironment.

In more general terms, nanoparticles have the potential to cross the BBB when decorated with specific ligands, such as proteins and antibodies, that, upon binding to their respective receptor, follow the receptor-mediated transcytosis pathway [20]. This pathway enables more efficient and higher cargo transportation across the BBB compared with other transport pathways [7,21]. Among others, transferrin, apolipoprotein, lactoferrin, and rabies virus glycoproteins have been studied as BBB-crossing surface ligands due to the high expression levels of their respective receptors, which are as follows: transferrin receptor, low-density lipoprotein receptor, lipoprotein receptors, and acetylcholine receptors [22,23]. For example, transferrin receptors are expressed on the endothelial cells of brain capillaries, with an extracellular receptor concentration of 0.13 fmol/µg cell protein; they are involved in iron transport to the brain via receptor-mediated transcytosis [24]. This pathway has been successfully exploited by transferrin-coated nanoparticles [20]. Recent studies show that transferrin-decorated nanoparticles can also promote a higher cellular uptake in brain cancer cells [25,26]. However, the nanoparticle surface ligand density differs between published studies, and it is often not well characterized. For example, a study compared liposomes with different surface transferrin antibody densities (0.15, 0.3, and 0.6 × 10^3^ antibodies µm^−2^), and surprisingly, it was shown that the nanoparticles with the highest density limited BBB crossing capabilities [27].

In addition, BBB transportation efficiency can be associated with the size of nanoparticles, but the mechanism behind it remains ambiguous. Smaller nanoparticles generally have greater BBB penetration capabilities compared with larger nanoparticles [28,29,30]; for example, 100 nm poly-(lactic-co-glycolic acid) nanoparticles displayed longer blood circulation times and greater BBB penetration capabilities than the larger (i.e., 200 and 800 nm) nanoparticles in an in vivo mouse study [31]. In contrast, some other studies report that the size of nanoparticles does not influence their BBB penetration capabilities [27,32,33]. And one in vitro study showed exactly the opposite trend; larger polystyrene nanoparticles (500 nm) had greater BBB penetration capabilities than smaller nanoparticles (200 nm) [34].

pSiNPs nanoparticles exhibit immense potential as candidates for drug delivery purposes due to properties such as their biodegradability, large surface area for drug loading, and biocompatibility [35]. Recent studies have also demonstrated the potential of using pSiNPs for glioblastoma multiforme cancer targeting after crossing the BBB [26,36]. However, a clearer understanding of the optimal nanoparticle size and transferrin surface content, in order to maximize BBB penetration capabilities, is still required. Therefore, a systematic comparison of pSiNPs nanoparticular features, such as surface density and size, with regard to BBB penetration, using well-defined assays, is urgently needed to advance the development of this drug delivery platform for the treatment of central nervous system diseases. Herein, we investigate the optimal surface and size influence of this delivery system for BBB penetration. Using bifunctional poly(ethylene glycol)-(PEG) linkers (with either carboxyl or methoxy terminal group), we were able to tune the transferrin density on pSiNPs. In addition, different sizes of nanoparticles were prepared using a centrifugation-based size selection method. This pSiNP library was then assessed for their cell association and cellular uptake by means of flow cytometry and confocal microscopy in 2D cell culture. Afterward, the pSiNPs’ transcytosis efficiency was investigated using both a static in vitro Transwell BBB model and a dynamic in vitro microfluidic BBB model (BBB-on-a-chip).

## 2. Materials and Methods

### 2.1. Materials

Silicon wafers used for the manufacturing of the nanoparticles were purchased from Siltronix (Archamps, France). Luminescent cell viability assay and VivoGlo^TM^ Luciferin were purchased from Promega. Cyanine5 amine (Cy5) was purchased from Lumiprobe. Hydrofluoric acid (HF, 49%) was purchased from J. T. Baker (Center Valley, PA, USA). Human holo-transferrin, undecylenic acid (UA), 1-ethyl-3-(3-dimethylaminopropyl)carbodiimide (EDC) hydrochloride, *N*-hydroxysulfosuccinimide (NHS), 2-(*N*-morpholino)ethanesulfonic acid (MES) hydrate, ethanol (EtOH), dimethylformamide (DMF), dichloromethane (DCM), and triethylamine (TEA) were purchased from Merck (Macquarie Park, Australia). All solvents were of analytical grade. Water (HPLC grade) was obtained with a Milli-Q Advantage A10 water purification system (Merck Millipore, Bayswater, Australia). α-Carboxyl-ω-amino poly(ethylene glycol) 10 kDa (NH_2_-PEG-COOH) and methoxy poly-(ethylene glycol)-amine 10 kDa (mPEG-NH_2_) were purchased from Advanced BioChemicals (Lawrenceville, GA, USA). All other chemicals were purchased from Sigma-Aldrich (Macquarie Park, Australia) unless stated otherwise.

### 2.2. Methods

#### 2.2.1. Attenuated Total Reflection-Fourier Transform Infrared Spectroscopy (ATR-FTIR)

ATR-FTIR spectra of all nanomaterials were obtained using a Thermo Scientific Nicolet 6700. A diamond crystal was run in ATR configuration with a 2 mm diamond tip and a deuterium triglycine sulfate detector. The spectra collected were averaged from 64 recorded scans with a resolution of 8 cm^−1^. Background spectra were blanked using air. The data were processed using OMNIC software (version 7.3).

#### 2.2.2. Transmission Electron Microscopy (TEM)

Bright Field (BF) TEM images were used to measure the nanoparticle size and visualize its morphological traits. TEM samples were prepared via drop casting the suspended sample of interest (in either ethanol or PBS) onto copper grids. The samples were examined using a Philips Tecnai 12 TEM at an operating voltage of 120 kV. Images were recorded using a FEI Eagle 4 k × 4 k CCD camera. Low dose conditions (<10 e^−^/Å^2^) were used to avoid damage to samples. 

#### 2.2.3. Dynamic Light Scattering (DLS)

A Malvern Instruments Zetasizer Nano instrument ZEN3600 was employed with a 4 mW 633 nm HeNe gas laser. An Avalanche photodiode detector measured the back scatter light at an angle of 173° relative to the angle of the incident light beam. Samples were dissolved in ethanol or PBS (pH 7.41). A single-use folded capillary cell commercialized by Malvern Instruments (model DTS 1070) was used for measuring surface zeta potential.

#### 2.2.4. Electrochemical Etching of Silicon Wafer

A highly boron-doped p-type silicon wafer (0.00055–0.001 Ω cm resistivity, 6-inch) was anodically etched in a solution composed of 3:1 (*v*:*v*) of 49% aqueous HF: ethanol. The etching waveform consisted of a square wave in which a lower current pulse of 0.6 A for 20 s was followed by a higher current pulse of 24 A applied for 0.2 s (the latter used to form sacrificial layers). This waveform was repeated for 1000 cycles. The film was then lifted off from the silicon substrate by applying a current density of 24 A for 60 s in a solution containing 1:1 (*v*:*v*) of 49% aqueous HF: ethanol [37]. The film was then stored in a desiccator. 

#### 2.2.5. Thermal Hydrocarbonization of Porous Silicon Film

The freshly etched pSi film was placed in a ceramic boat into a quartz tube under a stream of constant N_2_ flow at 2 L min^−1^ for 45 min at room temperature (RT). Then a 1:1 N_2_-acetylene mixture flow was introduced into the tube at RT for 15 min, with the quartz tube being placed into a preheated furnace at 525 °C for 14.5 min under a continuous flow of 1:1 N_2_-acetylene, followed by 30 s with N_2_ only. After that, the tube was allowed to cool down to RT under N_2_ flow [36]. The thermal hydrocarbonized silicon film was stored in ethanol and analyzed by ATR-FTIR.

#### 2.2.6. Fractioning of Silicon Wafer by Ultrasonication

The pSi film was shattered via shaking and then transferred into a 15 mm diameter Pyrex Quickfit glass test tube. The bottom of the test tube was filled with the shattered pSi film and then topped up with 6 mL ethanol. The mixture was then continuously ultrasonicated using a QSonica sonifier probe (Model CL-188) at 25% amplitude for 24 h in an ice bath with ice changed every 8 h. The thermal hydrocarbonized pSiNPs (THC-pSiNPs) were stored in ethanol [37].

#### 2.2.7. Size Selection and Functionalization of pSiNPs with Undecylenic Acid (UDA)

Size selection was carried out using a bespoke centrifuge-based protocol. First, the dispersion of nanoparticles was centrifugated at 2000× *g* for 15 min, and the supernatant was collected. This step was repeated once to remove all large nanoparticles. Afterward, the combined supernatants were centrifugated at 19,000× *g* for 45 min to remove the remains of the sacrificial layers. The remaining precipitates were then redispersed in ethanol and centrifugated at 3500× *g* for 15 min to further narrow the size distribution, and supernatants were kept. The last two steps were repeated once respectively to improve the yield of selected medium-sized (170–180 nm in diameter) particles. These are referred to as the small (S) pSiNPs throughout the manuscript.

The larger pSiNPs (L, around 403 nm diameter) were selected by collecting the pellets from the first two centrifugation steps (i.e., 2000× *g* for 15 min), redispersing these in ethanol, and then collecting the supernatants after centrifugation at 1000× *g* for 15 min to remove very large particles.

Afterward, these two size-selected particles were divided into several Eppendorf tubes and centrifugated at 21,000× *g* for 10 min to remove ethanol. Pellets were then dispersed in 2% HF in ethanol for 5 min to remove any oxidation and washed thoroughly with ethanol three times. Next, the dispersion was transferred into a 20 mL glass vial, sealed with a rubber septum, and dried under N_2_ flow. Next, 5 mL of undecylenic acid was prepared in another glass vial sealed with a rubber septum and sparged with N_2_ for 30 min in a warm water bath. The deoxygenated undecylenic acid was then transferred to the dried particle-containing glass vial via a cannula under N_2_ flow. The nanoparticles in undecylenic acid were sonicated to redisperse and reacted at 140 °C in an oil bath for 16 h [36]. Afterward, the unreacted undecylenic acid was removed by washing it with ethanol five times. The UDA-functionalized pSiNPs (UDA-pSiNPs) were stored in ethanol and analyzed via ATR-FTIR.

#### 2.2.8. Preparation of Cy5-Labeled Transferrin and PEG-Conjugated Nanoparticles 

Cy5, bifunctional PEG, and transferrin were all covalently conjugated to the carboxylic acid groups of the UDA-pSiNPs via EDC/NHS reaction. Briefly, pSiNPs were first washed three times with DMF to remove ethanol. Afterward, EDC and NHS (concentration of 10 mg/mL DMF) were added directly to UDA-pSiNPs (concentration of 4 mg/mL) with final concentrations of 10 mM and 5 mM, respectively. These reaction components were well mixed and allowed to react for 2 h at RT. After the NHS ester activation, the reaction mixture was washed with DMF and MES buffer to remove excess EDC and NHS. The activated UDA-pSiNPs were kept in low protein-binding tubes for direct surface conjugation.

For the two different sizes of NH_2_-PEG-COOH and transferrin-coated pSiNPs, Tf-PEG-pSiNPs(S) and Tf-PEG-pSiNPs(L), 3 µL of Cy5-NH_2_ (10 mg/mL in DMSO) and 500 µL of NH_2_-PEG-COOH (10 mg/mL) in phosphate-buffered saline (PBS, pH 8) were added to 1 mg of two different sizes of activated UDA-pSiNPs. The mixtures were sonicated for 0.5 h and agitated for 1 h at RT. Afterward, the reaction mixture was washed three times in DMF and once in PBS (pH 7.4) to remove any free Cy5 and PEG linker. To conjugate transferrin to Cy5-labeled nanoparticles, these samples were reactivated with EDC (2.5 mM) and NHS (1.25 mM) in 500 µL of MES buffer for 20 min at RT followed by two times washing in MES buffer and once in PBS (pH 7.4). Afterward, 500 µL of transferrin (2 mg/mL) in PBS (pH 7.4) was added to 1 mg of the reactivated nanoparticles, followed by sonication for 0.5 h and agitation overnight at RT. The final products were washed four times in PBS (pH 7.4) to remove unreacted and non-covalently associated transferrin, where supernatants were collected and measured using a bicinchoninic acid assay (BCA) assay [38] to indirectly determine the amount of transferrin conjugated onto the nanoparticles.

For two different ratios of NH_2_-PEG-COOH/mPEG-NH_2_ and transferrin-coated pSiNPs (Tf-PEG/mPEG(1:9)-pSiNPs(S) and Tf-PEG/mPEG(1:50)-pSiNPs(S), 3 µL of Cy5-NH_2_ (10 mg/mL in DMSO) and 50 µL or 5 µL NH_2_-PEG-COOH (10 mg/mL in PBS (pH 8) and 450 µL or 495 µL of mPEG (10 mg/mL) in PBS (pH 8) were added to 1 mg of activated UDA-pSiNPs, respectively. The mixtures were sonicated for 0.5 h and agitated for 1 h at RT. Afterward, these samples were treated using the same procedure in terms of transferrin conjugation and washing steps as mentioned above in the Tf-PEG-pSiNPs(S) and Tf-PEG-pSiNPs(L).

Finally, for mPEG-coated pSiNPs, briefly, 3.6 µL of Cy5-NH_2_ and 500 µL of mPEG-NH_2_ (10 mg/mL) in PBS (pH 8) were added to 1 mg of activated UDA-pSiNPs followed by sonicating for 0.5 h and agitating for 1 h at RT. Constantly sonicating and mixing the sample is the key step to fabricating mPEG-pSiNPs(S). Afterward, the reaction mixture was washed three times in DMF and one time in PBS (pH 7.4) to remove any free Cy5 and mPEG-NH_2_.

To prepare a positive control sample, transferrin-modified pSiNPs (Tf-pSiNPs), the procedure was followed from the previous report [36]. Briefly, 2.4 µL of Cy5-NH_2_ (10 mg/mL in DMSO) was added to 1 mg of activated UDA-pSiNPs in 500 µL of PBS (pH 8), sonicating for 0.5 h and agitating for 1 h at RT. Afterward, these samples were treated using the same procedure in terms of transferrin conjugation and washing steps as mentioned above in Tf-PEG-pSiNPs(S) and Tf-PEG-pSiNPs(L).

#### 2.2.9. Cell Culture

Immortalized human brain microvascular endothelial cells (hCMEC/D3) were purchased from Merck. The hCMEC/D3 cell line was maintained with endothelial cell growth basal medium-2 (EBM-2, Lonza) with supplements of 5% fetal bovine serum (FBS) and growth factors as previously reported [39]. Cells were cultured on a collagen-coated (150 µg/mL) T-75 tissue culture flask at 37 °C in 5% CO_2_ in the passage of 28 to 35 according to the supplier’s protocol. The cell culture medium was changed every 2 to 3 days before reaching confluency. The cell culture procedure in the BBB-on-a-chip model was followed as previously reported [40].

#### 2.2.10. Cell Viability in Contact with pSiNPs

The biocompatibility of modified pSiNPs on the hCMEC/D3 cell line was determined using a CellTiter-Glo^®^ luminescent cell viability assay (Promega, Alexandria, Australia). Briefly, hCMEC/D3 cells were seeded onto a 96-well white opaque polystyrene microplate (Sigma-Aldrich, Macquarie Park, Australia) at a density of 10,000 cells per well and maintained in the cell culture medium for 1 day. Subsequently, cells were treated with different concentrations (5, 10, 25, 50 µg/mL) of surface-modified pSiNPs. Cells without any treatment were used as control, and each condition was triplicated. After incubating cells for 48 h, a CellTiter-Glo^®^ luminescent cell viability assay was used to evaluate the cell viability. Briefly, 100 µL of the solution of the assay kit was added to 100 µL of the medium in each well, and the plates were gently shaken at RT for 15 min. Finally, the luminescence intensity of each sample was obtained using a PerkinElmer EnSpire multimode plate reader, and data were expressed as the mean and standard deviation of 3 replicates.

#### 2.2.11. Cellular Association and Uptake of pSiNPs via Flow Cytometry and Confocal Microscopy

Flow cytometry was used to confirm the cellular association of pSiNPs in hCMEC/D3. Briefly, hCMEC/D3 was seeded onto a 24-well plate at the density of 1.2 × 10^5^ cells per well and cultured overnight. The cells were then washed twice in PBS and incubated with 5 µg/mL of Cy5-labeled surface-modified pSiNPs for 1 h at 37 °C supplied with 5% CO_2_. Afterward, the cells were washed 3× with PBS to remove any unattached pSiNPs, and the cells were harvested via trypsin and centrifugation for 3 min at 180× *g*. The resulting cell pellets were dispersed in cold PBS and stained with propidium iodide (PI) (5 µg/mL) for 5 min to assess cell viability. Samples were then analyzed using flow cytometry (BD FACS Canto II) for Cy5 and PI fluorescence signals. Cellular association percentage was calculated as the number of cells that displayed fluorescence signals compared to untreated cells (Cy5 negative).

For visualizing the cellular association and uptake, confocal microscopy was used. hCMEC/D3 cells were seeded onto an 8-well chamber (Ibidi) at the density of 1 × 10^5^ cells per well and allowed to attach and were cultured for 1 day. The cells were washed twice in PBS and then treated with Cy5-labeled surface-modified pSiNPs (5 µg/mL). After 1 h, the wells were washed twice in PBS and fixed in 4% paraformaldehyde (PFA) for 15 min at RT followed by permeabilization with 0.1% Triton-X-100 in PBS for 5 min RT. The cells were then washed twice using PBS and incubated with Hoechst 33342 (5 µg/mL, Thermo Fisher Scientific, Scoresby, Australia) and Alexa Fluor™ 488 Phalloidin (5 µg/mL Thermo Fisher Scientific, Scoresby, Australia) for 30 min. Afterward, the cells were washed three times with PBS, and the images were taken using confocal microscopy (Leica TCS SP8, Leica Microsystems, Macquarie Park, Australia). Fluorophores were excited via 405, 561, and 647 nm laser lines. The laser powers and gains for each channel were adjusted against untreated controls to minimize sample autofluorescence.

#### 2.2.12. BBB Transwell Model Preparation for Nanoparticles Assessment

Transwell inserts (3 μm pore size, polyester membrane, Sigma-Aldrich, Macquarie Park, Australia) were first coated with 100 µL collagen-I (Sigma-Aldrich, Macquarie Park, Australia) in a concentration of 150 µg/mL in the incubator for 1 h and were seeded with hCMEC/D3 at a density of 1.65 × 10^5^ cells/mL in 200 µL of cell medium. The bottom compartment was filled with 600 µL of cell medium. The cell culture medium was changed every two days until a monolayer formed. The transendothelial electrical resistance (TEER) was assessed every day for 7 to 10 days using a Millicell ERS-2 voltammeter EVOM2 and an STX02 chopstick electrode (Merck Millipore, Bayswater, Australia).

FITC-dextran (10 kDa) was used to confirm the BBB integrity formed on the Transwell culture insert after 7 days. Briefly, 200 µL of FITC-dextran (50 µg/mL) in complete cell culture media was added to the top compartment with a bottom compartment filled with 600 µL of complete culture media. Inserts were then incubated at 37 °C, 95% humidity, and 5% CO_2_ for 6 h. A 200 µL aliquot was collected every hour to measure FITC fluorescence signals in the microplate reader, which was replaced with fresh 200 µL of complete cell culture medium. The concentration of dextran at each time point was calculated using a standard curve of dextran. The apparent permeability coefficients (*P_app_*), as the indication of the integrity of BBB, were calculated according to the following equation [41]:

Papp=dC/dt·Vr/(A·C)

where *Vr* (mL) is the volume of the lower compartment, *dC*/*dt* is the slope of the cumulative concentration of the dextran in the lower compartment over time, *A* (cm^2^) is the surface area of the inset and *C* (µg/mL) is the initial concentration of dextran that was placed into the top compartment [34].

To investigate the BBB barrier integrity through examination of the cell surface protein expression levels, a confluent monolayer of brain endothelial cells was cultured in an 8-well Nunc Lab-Tek II Chamber Slide System. This confluent monolayer barrier was washed with PBS twice and incubated with 4% paraformaldehyde (PFA) at RT for 10 min. Fixed cells were then washed with PBS to remove PFA and permeabilized with 0.2% Triton X-100 in PBS for 10 min. Permeabilized cells were then washed with PBS three times and blocked with 2.5% BSA for 60 min at RT. After blocking, the cells were washed with PBS three times and incubated with anti ZO-1 (Cell Signaling Technology, Danvers, MA, USA D6L1E, rabbit mAb) (1:100 primary antibody diluted in 2.5% BSA PBS buffer) at 4 °C overnight. Cells were washed with PBS 2× before the secondary antibody goat anti-rabbit Alexa Fluor 488 antibody (Thermo Fisher, Scoresby, Australia) was added and incubated for 1 h at RT. Finally, the slides were rinsed with PBS 3×, and ProLong Diamond Antifade Mountant with DAPI (Thermo Fisher, Scoresby, Australia) was added. Samples were cured for 2 h at RT in the dark and tight junction expressions were imaged using a confocal fluorescence microscope (Leica TCS SP8, Leica Microsystems, Macquarie Park, Australia).

For assessment of nanoparticle performance in Transwells, on day 7 of monolayer culturing, diverse types of pSiNPs (50 µg/mL) were incubated at the top compartment (in 200 µL completed culture medium) and the lower compartment was filled with 600 µL of complete culture media and further incubated at 37 °C and 5% CO_2_ for 48 h. After that, a 200 µL aliquot was collected from the bottom compartment and measured via the fluorescence microplate reader for the fluorescence intensity (assessing the Cy5 signal). The concentration was determined using a calibration curve of different concentrations of nanoparticles in the cell medium. The percentage of nanoparticles in the bottom compartment was calculated as the fluorescence intensity from the bottom compartment was divided by the original fluorescence intensity of nanoparticles that were placed in the top compartment.

#### 2.2.13. BBB-on-a-Chip Model Establishment and Nanoparticles Assessment

The BBB-on-a-chip models were prepared as described previously [40]. Briefly, the design includes three main channels (blood/brain/medium). Each channel is 500 µm wide, 100 µm high, and 2.0 cm in length. An array of microchannels (3 µm width, 80 µm length, and 3 µm height) connects the blood channel with the brain channel. Additional microchannels (50 µm in width, 80 µm length, and 3 µm height) are present between the brain channel and medium channel to facilitate the supply of nutrients.

After cells formed a monolayer in the blood channel, Matrigel (Corning, Mulgrave, Australia) was added to the brain channel to facilitate visualization of nanoparticles that cross the blood channel. Three control experiments were conducted before the nanoparticle assessment. Firstly, after forming a cell monolayer in the blood channel of BBB-on-a-chip, cells were subjected to immunofluorescence analysis (same procedures as mentioned before for the Transwell protein expression) using a confocal microscope (Leica TCS SP8, Leica Microsystems, Macquarie Park, Australia). Secondly, 10 kDa FITC-dextran (25 µg/mL) was diluted in EBM-2 medium and flowed through the blood channel in cell-seeded chips and blank chips (without cells seeded) at a flow rate of 5 µL/min using a programmable syringe pump (Harvard Apparatus, Inc, Holliston, MA, USA.). Afterward, these chips were subjected to the confocal microscope (Leica TCS SP8, Leica Microsystems, Macquarie Park Australia) to quantify the permeation of FITC-dextran to the brain channel. Lastly, Tf-pSiNPs were diluted in EBM-2 medium to a concentration of 10 µg/Ml and flowed through chips in which the blood channel (previously seeded with cells or left empty). Afterward, these chips were rinsed with PBS and subjected to confocal microscopy.

To assess nanoparticles in BBB-on-a-chip, nanoparticles and 10 kDa FITC-dextran were diluted in EBM-2 medium to the concentrations of 10 µg/Ml and 25 µg/Ml, respectively, and flowed through the blood channel at a flow rate of 5 µL/min using a programmable syringe pump (Harvard Apparatus) for 4 h. The BBB-on-a-chip models were kept inside an incubator (37 °C supplied with 5% CO_2_). After stopping the flow, the chips were detached from the syringe pump and carefully rinsed with PBS to remove any unbonded nanoparticles. Afterward, cells in the chips were fixed with 4% PFA, stained with DAPI, and subjected to confocal microscope (Leica TCS SP8, Leica Microsystems, Macquarie Park, Australia). FITC-dextran penetration in each sample was assessed. All experiments were triplicated using independent BBB-on-a-chip. The relative fluorescence intensity of nanoparticles in the microchannels of chips was further analyzed using ImageJ (NIH, Bethesda, MD, USA) and plotted using GraphPad Prism 7.

## 3. Results and Discussions

### 3.1. Preparation of pSiNPs with Various Ligand Surface Densities and Sizes

In brief, the pSiNPs were fabricated as followed. Initially, a multilayered porous silicon film was prepared through electrochemical etching of a silicon wafer in an ethanolic HF solution, followed by thermal hydrocarbonization (THC) [36,42]. This surface modification was conducted to enhance the stability of the pSiNPs and prevent any particle degradation during in vitro experiments which might complicate data analysis. ATR-FTIR spectroscopy confirmed the successful THC modification, as demonstrated by the existence of a C-H stretching absorption band from 2800 to 3000 cm^−1^ and a Si-C band at 1063 cm^−1^ (Figure S1). The THC-modified porous silicon film was then fractured into THC-pSiNPs through ultrasonication. Two different sizes of pSiNPs, referred to as small (S) and large (L), were subsequently prepared using a bespoke centrifuge-based size selection protocol.

Transmission electron microscope (TEM) images revealed that THC-pSiNPs(S) had an irregular, plate-like shape [37], with an average particle size of 199.8 ± 40.3 nm in length and 86.3 ± 12.6 nm in thickness, along with a pore size of 21.8 ± 5.3 nm. The average hydrodynamic diameter, as determined via dynamic light scattering (DLS), was 180.9 ± 63.2 nm, with a relatively low polydispersity index (PDI) of 0.11 (Figure S2). The larger porous silicon nanoparticles, THC-pSiNPs(L), exhibited an average particle size of 427.4 ± 144.2 nm in the x-y dimension, 177.6 ± 83.2 nm in the z dimension, and a pore size of 26.8 ± 7.5 nm. Their average hydrodynamic diameter was 291.6 ± 88.4 nm, with a PDI of 0.22 (Figure S2). The inconsistency between DLS and TEM measurements can be attributed to the plate-like shape of the particles.

The surface of the two types of nanoparticles was then modified with functional carboxyl groups through hydrosilylation of surface silicon hydride groups with 1-undecylenic acid (UDA) to generate UDA-pSiNPs (Figure S1) [43]. ATR-FTIR spectra confirmed this surface modification as demonstrated by the emergence of a C=O band at 1716 cm^−1^ attributed to the carbonyl bond in UDA. Furthermore, more pronounced absorption bands emerged between 2800 cm^−1^ and 3000 cm^−1^ corresponding to C-H stretching vibrations from the aliphatic chains in UDA (Figure S1). The UDA modification did not significantly alter particle or pore size, as confirmed via TEM and DLS (Figure S2).

**Figure 1:**
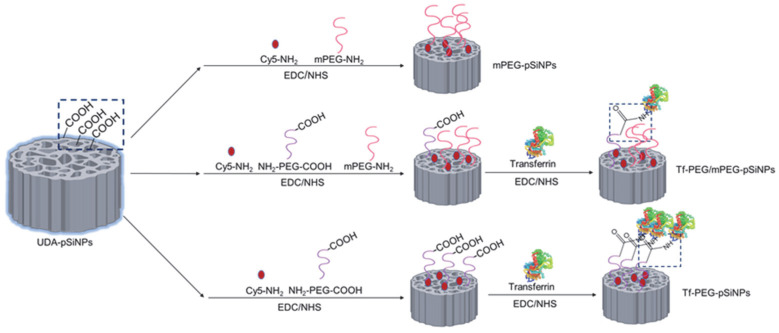
Schematic diagram of fabrication of a nanoparticle library with different transferrin ligand densities using different ratios of mPEG-NH_2_ and NH_2_-PEG-COOH.

As shown in Figure 1, UDA-pSiNPs were conjugated with varying ratios of mPEG-NH_2_ and NH_2_-PEG-COOH (with an average molecular weight 10 kDa). This conjugation process aimed to fabricate pSiNPs with distinct surface densities of the functional carboxyl group. Simultaneously, the pSiNPs were co-labeled with Cy5 fluorophores to enable direct comparison between the nanoparticles in subsequent assays. Following activation of the carboxylic groups with NHS, an excess of transferrin was added to ensure maximal conjugation. This led to the creation of a library of Cy5-labeled PEGylated pSiNPs featuring diverse transferrin surface densities. Notably, the larger nanoparticle, pSiNP(L), was only modified with NH_2_-PEG-COOH.

**Figure 2:**
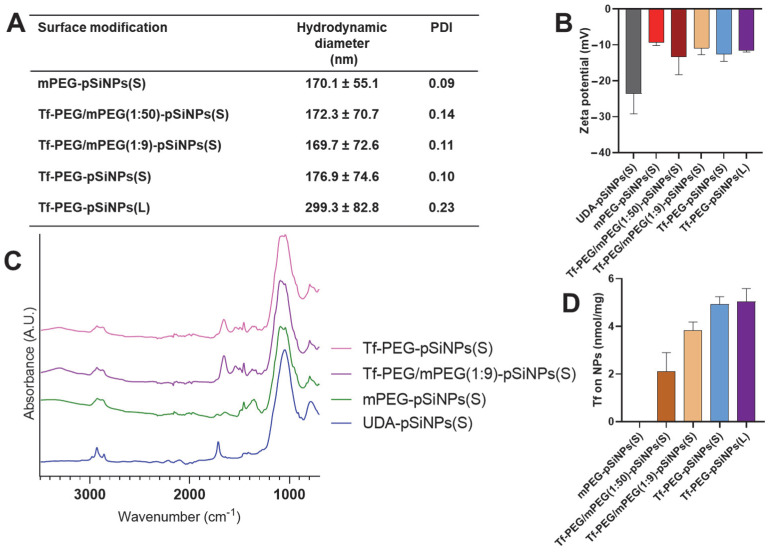
Physicochemical characterization of different surface-modified pSiNPs. (**A**) Particle hydrodynamic diameter (z-average) and PDI measurements by DLS; (**B**) ζ-potential; (**C**) ATR-FTIR spectra; and (**D**) transferrin quantification of pSiNPs based on BCA assay. Data in (**B**,**D**) are shown as mean ± standard deviation, N = 3. The two different sizes of pSiNPs are referred to as small (S) and large (L).

ATR-FTIR was used to investigate the success of these modification steps (Figure 2C and Figure S3). For ease of comparison, all spectra were normalized to the Si-C/Si-O signal (1000–1100 cm^−1^). After modification with either mPEG-NH2 or NH2-PEG-COOH, a distinct vibration band appeared at 1100 cm^−1^ (C-O) adjacent to the Si-C/Si-O signal (1000–1100 cm^−1^). Moreover, stronger signals from C-H stretching bands in the 2800 to 3000 cm^−1^ range were present too. Compared to mPEG-modified pSiNPs (mPEG-pSiNPs(S)), the transferrin-modified pSiNPs exhibited more pronounced C=O amide bands at 1657 cm^−1^, indicating the successful conjugation of the amide-rich transferrin (Figure 2C). Moreover, the absence of the carboxyl C=O signal at 1716 cm^−1^ from UDA indicated successful amidation of PEG-COOH with transferrin. After transferrin and mPEG-NH2 modification, the edges of these nanoparticles appeared less distinct—suggesting the presence of an organic coating, i.e., PEG and transferrin (Figures S4 and S5). DLS showed that the transferrin and PEG-modified pSiNPs(S) maintained a similar hydrodynamic diameter of around 170.1 ± 55.1 nm with low PDI values ranging from 0.07 to 0.11 (Figure 2A). Conversely, the larger nanoparticles (Tf-PEG-pSiNPs(L)) exhibited a hydrodynamic diameter of 299.3 ± 82.8 nm. Importantly, the PEG modification significantly improved the colloidal stability of the nanoparticles, as UDA-pSiNPs(S) precipitated immediately in water.

The amount of transferrin in different samples was determined indirectly using a BCA assay, measuring unconjugated transferrin present in the supernatant that was collected during the washing steps (Figure 2D). The amount of conjugated transferrin for the three different NH_2_-PEG-COOH and mPEG-NH_2_ ratios (1:50, 1:9, and 1:0)—represented as Tf-PEG/mPEG(1:50)-pSiNPs(S), Tf-PEG/mPEG(1:9)-pSiNPs(S), Tf-PEG-pSiNPs(S) and Tf-PEG-pSiNPs(L))—was determined, using the BCA assay, to be 2.10 ± 0.79, 3.83 ± 0.34, 4.92 ± 0.31, 5.03 ± 0.54 nmol per mg of nanoparticles, respectively. This unit (nmol per mg of nanoparticles) was used to express the ligand surface density, avoiding potential inaccuracies arising from attempting to convert it to a number of ligands per nanoparticle due to the porous and irregular morphology of the pSiNPs. These calculated transferrin amounts were as expected as the number of functional carboxyl groups available for transferrin conjugation increased for each sample. Notably, the larger transferrin-coated nanoparticles (Tf-PEG-pSiNPs(L) displayed a similar transferrin amount (around 5 nmol per mg of nanoparticles) compared to the smaller Tf-PEG pSiNPs(S), as both particles possess similar surface areas due to their inherent porosity. The ζ-potential of solely mPEG-coated pSiNPs was less negative than that of UDA-pSiNPs(S) (−20 mV) due to the neutral charge of the mPEG linker upon conjugation (Figure 2B). Some residual negative charge was expected as not all carboxyl groups can react, due to steric hindrance caused by the PEG linker (10 kDa). All transferrin-PEG-modified samples also showed slightly negative surface charges, ranging range from −10 mV to −15 mV. These charges resulted from the combination of neutral mPEG, the slight negative charge of transferrin, and potentially remaining surface carboxyl groups, as described above.

### 3.2. Evaluation of pSiNPs in Brain Microvascular Endothelial Cells

The brain microvascular endothelial cells (hCMEC/D3) were incubated with varying concentrations of modified pSiNPs for 48 h, followed by assessment using an ATP activity-based luminescence assay (Figure S5). Across all samples, no significant toxicity was observed up to a concentration of 50 µg/mL. At the highest concentration of 50 µg/mL for some of the pSiNPs samples, a slight decrease in cell viability was noted, ranging from 83.6 ± 12.6% to 89.0 ± 1.7%. Furthermore, the permeability of the hCMEC/D3 monolayer in Transwell assays was not affected after incubation with 50 µg/mL of modified pSiNPs for 48 h, as determined by their TEER values (described in Section 3.3). Hence, we carried out all in vitro assays using concentrations no higher than 50 µg/mL.

**Figure 3:**
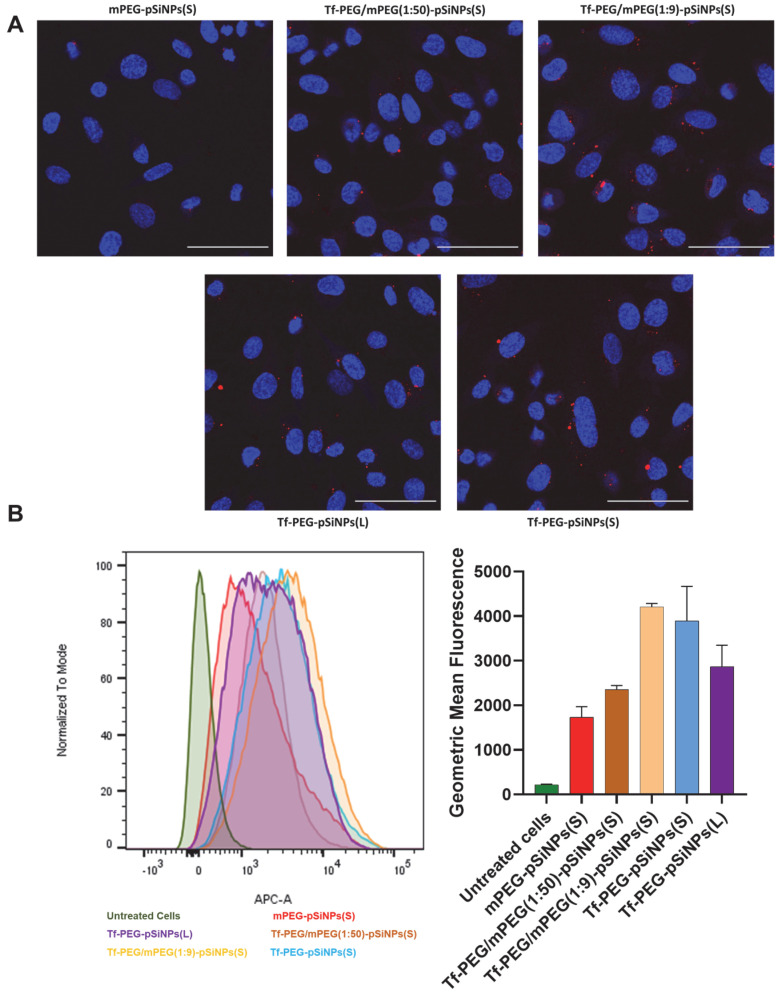
(**A**) Confocal fluorescence microscopy images of hCMEC/D3 cells after incubation with mPEG-pSiNPs(S), Tf-PEG/mPEG(1:50)-pSiNPs(S), Tf-PEG/mPEG(1:9)-pSiNPs(S), Tf-PEG-pSiNPs(L) and Tf-PEG-pSiNPs(S) (blue = nucleus, green = F-actin, red = Cy5 in nanoparticles, scale bars = 50 µm); (**B**) flow cytometry analysis of hCMEC/D3 cellular association of different modified pSiNPs. Histogram and the geometric mean of Cy5 positive hCMEC/D3 cells by treating different modified samples. The geometric mean values are shown by mean ± sd, N = 3.

Confocal microscopy and flow cytometry were used to investigate the interaction of different surface-modified nanoparticles with hCMEC/D3 cells. It is important to note that all pSiNP samples exhibited similar Cy5 intensities on a per mass basis, allowing for direct comparisons in subsequent measurements (Figure S6). The confocal images of particle association and uptake are presented in Figure 3A. Notably, all transferrin-conjugated nanoparticles showed higher Cy5 fluorescence signals within the cells compared to solely mPEG-modified particles. This finding is in alignment with previous reports illustrating the role of transferrin in nanoparticle association with brain endothelial cells. Our prior study, for instance, demonstrated that BSA-coated pSiNPs showed a lower cell association than Tf-coated pSiNPs and that Tf-coated pSiNPs were predominantly taken up via clathrin and caveolae-mediated endocytosis [44]. Nanoparticle internalization and uptake into the cells were confirmed by z-stack scanning confocal microscopy (Figure S7). These images showed that Tf-PEG-pSiNPs(S) were located within the cells, providing evidence of successful uptake and internalization.

Flow cytometry was employed to quantitatively assess the impact of transferrin surface density on cell association in hCMEC/D3 cells (Figure 3B). All transferrin-modified nanoparticles presented higher geometric fluorescence mean intensity (GMF) in comparison to the mPEG-pSiNPs(S) sample, consistent with the observations from confocal imaging. Furthermore, differences in cell association were discernable among nanoparticles with varying transferrin surface densities. Although the lowest transferrin surface density, Tf-PEG/mPEG(1:50)-pSiNPs(S), displayed only a slightly higher cellular association than mPEG-pSiNPs(S), Tf-PEG/mPEG(1:9)-pSiNPs(S) (boasting a higher ligand density) showed a significant increase, approximately doubling the GMF compared to Tf-PEG/mPEG(1:50)-pSiNPs(S) and mPEG-pSiNPs(S). Interestingly, no further increase in cell association was observed with the highest transferrin ligand density (Tf-PEG-pSiNPs(S)). This suggests a non-monotonic positive relationship between the range of transferrin surface density and its relative association and uptake for our pSiNP system. It is important to note that this phenomenon extends beyond pSiNPs; for example, Song et al. reported that higher ligand densities did not necessarily lead to increased uptake for transferrin-decorated micelles [45]. In addition, the larger Tf-PEG-pSiNPs(L) exhibited a notably lower GMF than Tf-PEG-pSiNPs(S) despite a similar transferrin coverage. This difference in GMF indicates that the size of nanoparticles also plays a critical role in the cellular association and uptake.

### 3.3. Assessment of pSiNPs Using Transwell Assay

**Figure 4:**
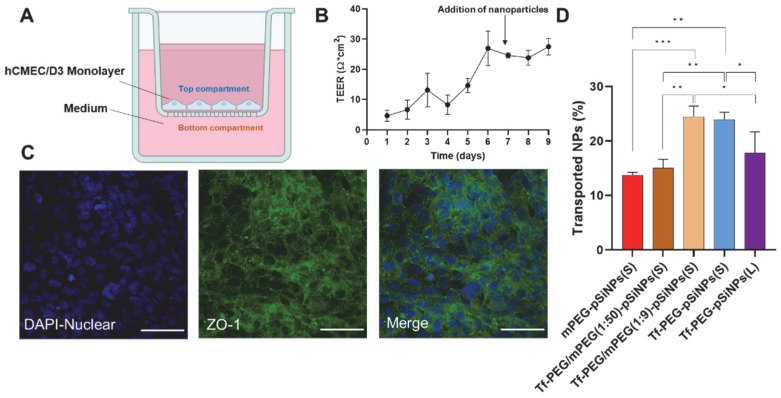
(**A**) Schematic representation of a BBB Transwell model that was formed by a monolayer of hCMEC/D3 cells over an insert with 3 µm sized pores; (**B**) TEER value of hCMEC/D3 monolayer over 9 days of culture, N = 3. The TEER value increased over time and reached a plateau on day 6. pSiNPs were applied to the Transwell insert on day 7; (**C**) Characterization of ZO-1 expression by the hCMEC/D3 monolayer (scale bar = 50 µm); and (**D**) percentage of accumulated nanoparticles in the bottom compartment of the Transwell after applying nanoparticles in the top compartment for 48 h, N = 3, one-way ANOVA test, * *p* < 0.1, ** *p* < 0.01, *** *p* < 0.001. All data are shown by mean ± sd.

The hCMEC/D3 transcytosis of the pSiNPs was first investigated using a Transwell model (Figure 4A). This model comprises two compartments, with an hCMEC/D3 monolayer cultured on an insert that separates the two compartments. The TEER value, a marker for BBB barrier tightness, was measured daily to confirm the presence of a confluent monolayer (Figure 4B). TEER values reached approximately 10 Ω × cm^2^ on day 4, increasing to around 26 Ω × cm^2^ by day 6. Afterward, TEER values remained stable, ranging between 25 and 27 Ω × cm^2^, comparable to literature values [34]. We also confirmed the presence of the tight-junction-associated protein, ZO-1, which is mostly expressed at the interface of cell-to-cell contact [34] (Figure 4C). The integrity of the BBB in the Transwell model was further assessed using 10 kDa FITC-labeled dextran. This yielded an apparent permeability value of 2.37 ± 0.38 × 10^−6^ cm/s (N = 3) on day 6, consistent with findings in other studies [46]. The TEER values obtained, the expression of ZO-1 protein, and the low permeability of FITC-dextran collectively affirm the integrity of the BBB formed on the Transwell inserts. As a control experiment, blank Transwells with inserts devoid of an hCMEC/D3 monolayer were treated with transferrin-coated pSiNPs to examine nanoparticle diffusion through the porous membrane without a functional BBB. This control revealed that 69–74% of the nanoparticles accumulated in the bottom compartment after 48 h, consistent with free diffusion of pSiNPs across both compartments, considering the volume of the bottom compartment accounted for 75% of the total volume. This control experiment also confirmed that the nanoparticles did not interact with the insert. Consequently, this Transwell model proved suitable for evaluating the permeability and thus transcytosis potential of the Cy-5 labeled nanoparticle library in an hCMEC/D3 monolayer.

All five nanoparticle types (mPEG-pSiNPs(S), Tf-PEG/mPEG(1:50)-pSiNPs(S), Tf-PEG/mPEG(1:9)-pSiNPs(S), Tf-PEG-pSiNPs(S), and Tf-PEG-pSiNPs(L)) were evaluated using the Transwell assay, and each experiment was done in triplicate. Among these, mPEG-pSiNPs(S) and Tf-PEG/mPEG(1:50)-pSiNPs(S) displayed the lowest average transport percentage (13.7% and 15.0%) to the bottom compartment. This is in accordance with them displaying their lowest association in the previous flow cytometry experiment. Tf-PEG/mPEG(1:9)-pSiNPs(S) and Tf-PEG-pSiNPs(S) both showed higher average accumulations of 24.4% and 23.6%, respectively, with no significant differences between them.

Transferrin-modified nanoparticles can cross the BBB by binding to transferrin receptors on endothelial cells. This binding initiates a process known as receptor-mediated transcytosis, which involves three key steps: (i) attachment of protein-coated nanoparticles to receptors on the endothelial cells, (ii) internalization and sorting of the nanoparticles within the cell, and (iii) release of the nanoparticles on the other side of the endothelial cells. Intuitively, one might expect that higher levels of transferrin on the nanoparticle surface would lead to a greater likelihood of attachment to the cells and BBB crossing. However, in the Transwell assay, nanoparticles with varying transferrin amounts, specifically, 3.83 ± 0.34 nmol per mg (Tf-PEG/mPEG(1:9)-pSiNPs(S)) and 4.92 ± 0.31 nmol per mg (Tf-PEG-pSiNPs(S)) presented similar transport levels. This reveals that there may be an optimal surface ligand density on pSiNPs, and an increase in transferrin surface density on the nanoparticles might not necessarily increase or could even diminish their penetration ability across the BBB. This could be caused by several factors: a higher transferrin density might result in an overly strong affinity to transferrin receptors, potentially preventing release [47] or possibly inducing lysosomal sorting and degradation, thus preventing transportation [48]. Interestingly, similar trends have been reported in nanoparticle delivery systems, where an excessively high ligand density hindered BBB penetration [27,49]. For example, gold nanoparticles with excessively high transferrin density exhibited reduced entry into the brain parenchyma in mice [49]. Similarly, liposomes with the lowest density of antibody exhibited comparable low levels of BBB transport as nanoparticles with the highest density of antibody, while the intermediate-density antibody nanoparticles exhibited the highest accumulation [27]. Our result demonstrated that the pSiNP delivery system shares some universal characteristics with other nanoparticles system. Specifically, an optimal surface ligand density appears crucial for efficient BBB penetration of pSiNPs.

The effect of the pSiNP size on BBB penetration was investigated by comparing two different sized nanoparticles with comparable ligand density, i.e., Tf-PEG-pSiNPs(L) (around 420 nm) and Tf-PEG-pSiNPs(S) (around 203 nm). Tf-PEG-pSiNPs(L) presented an average transport percentage of 17.5 ± 3.9%, while the smaller Tf-PEG-pSiNPs(S) showed a significantly higher average transport percentage of 23.6 ± 1.4%. This result aligns with their performance in cellular association and uptake in 2D cell culture experiments, suggesting that an increase in pSiNP size, while maintaining the same surface ligand density, can have a detrimental effect on BBB transport. Despite the unique physical structure of pSiNPs (porous and irregular), this outcome is consistent with observations in other nanoparticle systems [30,50].

### 3.4. Assessment of pSiNPs Using BBB-on-a-Chip Model

**Figure 5:**
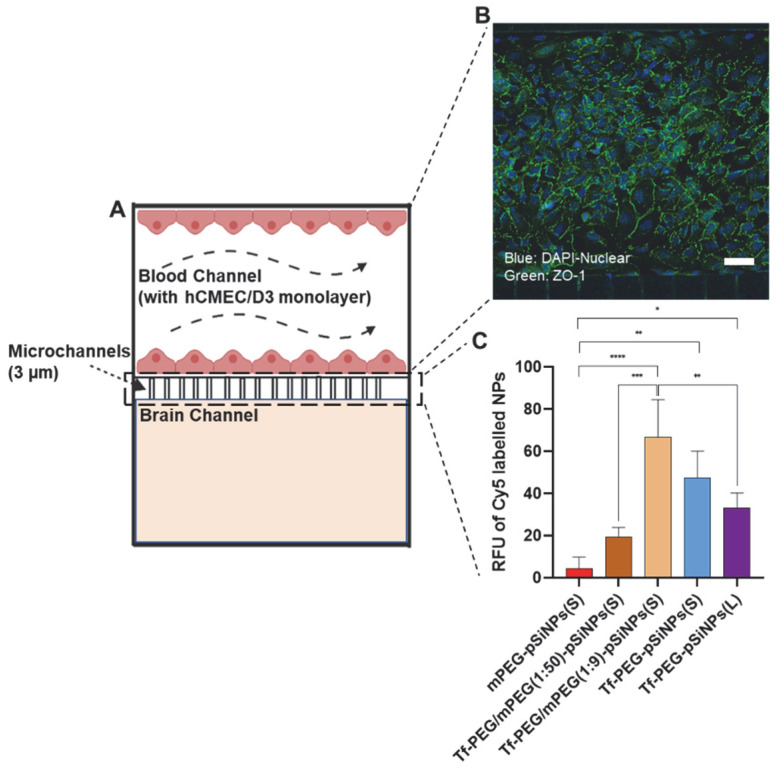
(**A**) Schematic of the BBB-on-a-chip model consisting of two channels. The blood channel was formed by a monolayer of hCMEC/D3 cells over 3 µm microchannels under a fluidic environment. (**B**) Characterization of ZO-1 expression by the hCMEC/D3 monolayer (scale bar = 50 µm). (**C**) Corresponding RFU of pSiNPs crossing blood channel in BBB-on-a-chip. N > 3, one-way ANOVA test, * *p* < 0.1, ** *p* < 0.01, *** *p* < 0.001, **** *p* < 0.0001. The values are shown by mean ± sd.

Although the Transwell assay is commonly used to assess nanoparticle transcytosis potential, it lacks the presence of hemodynamic shear stress that plays an essential role in endothelial cell phenotype and function. This factor is essential for achieving a more accurate physiological representation [51,52]. Moreover, flow conditions also influence the binding of nanoparticles to receptors and, consequently, cellular uptake [53]. Hence, we wanted to investigate if this factor would affect the trends observed for our set of pSiNPs. To address this, a dynamic model known as a BBB-on-a-chip was used to examine how flow affects the ability of nanoparticles to penetrate the BBB (Figure 5A) [40]. In the model, two main channels were constructed, representing the “brain” and “blood” compartments. The blood channel was constructed as a simplified version of the BBB, while the brain channel was used to assess nanoparticle penetration. Several validation experiments were performed to confirm the presence of a confluent physical monolayer on the chip. First, like the Transwell model, clear protein expression of ZO-1 was observed at the intercellular junctions of the seeded hCMEC/D3 chips, indicating the presence of tight connections in the monolayer (Figure 5B). Second, the integrity of the formed BBB layer was examined using 10 kDa FITC-labeled dextran, with a characteristic permeability value of 5.61 ± 1.48 × 10^−6^ cm/s (N = 3) [40]. Confocal microscopy further confirmed these findings as a much lower dextran fluorescence intensity was observed in the brain channel of the chips where the blood channel was seeded with cells compared to blank chips (Figure S8A,B). The average relative fluorescence intensity value (RFU) of randomly selected regions of interest in the brain channel was four times lower in seeded chips compared to blank chips (Figure S8C). In addition, we compared the penetration of nanoparticles in seeded BBB-on-a-chip versus blank chips, and as expected, significantly fewer nanoparticles were able to cross to the brain compartment in a seeded BBB-on-a-chip (Figure S9).

As a quality control measure, all microfluidic chips used for the assessment of the nanoparticle library were treated with FITC-dextran to ensure the presence of a functional barrier before experimentation (Figure S8D). Although the overall trend for the nanoparticle penetration remained consistent with the results from the Transwell assay, some intriguing differences emerged. mPEG-pSiNPs(S) and Tf-PEG/mPEG(1:50)-pSiNPs(S) again showed the lowest and second-lowest RFU (i.e., BBB crossing potential) (Figure 5C). In contrast, Tf-PEG/mPEG(1:9)-pSiNPs(S) presented the highest RFU, followed by Tf-PEG-pSiNPs(S) and Tf-PEG-pSiNPs(L).

The impact of the various transferrin surface densities was more pronounced in the BBB-on-a-chip model compared to the Transwell assay. For example, a clear increase in BBB penetration could be observed for nanoparticles with the lowest transferrin ligand density (Tf-PEG/mPEG(1:50)-pSiNPs(S)) when compared to mPEG-pSiNPs(S). This increase was not as apparent in the Transwell assay. Moreover, whereas the Transwell assay showed a 67% improvement in BBB crossing for nanoparticles with intermediate transferrin ligand density (Tf-PEG/mPEG(1:9)-pSiNPs(S)) when compared to those with low transferrin ligand density (Tf-PEG/mPEG(1:50)-pSiNPs(S)), the BBB-on-a-chip model demonstrated a much higher increase of approximately 300%. Of particular interest, in the BBB-on-a-chip model, nanoparticles with the highest transferrin density, Tf-PEG-pSiNPs(S), showed nearly a 40% lower BBB crossing potential compared to those with intermediate transferrin density (Tf-PEG/mPEG(1:9)-pSiNPs(S)). In contrast, these two variants had a similar BBB penetration in the Transwell assay. This confirms that the highest transferrin density on the pSiNPs does not necessarily translate to the highest BBB penetration. Notably, this observation becomes more prominent under the physiologically relevant conditions of the BBB-on-a-chip model. The observed differences between the Transwell and BBB-on-a-chip assays may be attributed to the influence of fluid flow on nanoparticle association and consequent transcytosis. Lin et al. investigated the difference in human aortic endothelial cellular association with nanoparticles, comparing those with targeted moieties (i.e., platelet glycoprotein Ibα), to those without, under both flow and static environments [54]. In their study, the cellular uptake of nanoparticles with targeting moieties was significantly higher under flow conditions than those without targeting moieties. Similarly, Zukerman et al. demonstrated that under shear stress, nanoparticles with higher ligand surface coating density exhibited significantly increased adhesion when compared to nanoparticles with lower ligand surface densities [55]. However, it is noted that the range of ligand densities on nanoparticles plays an important role in their cellular association. In some cases, an excessive number of ligands on the nanoparticle surfaces can hinder their selectivity and adhesion [56].

The BBB crossing efficacy of the two differently sized pSiNPs (Tf-PEG-pSiNPs(S) and Tf-PEG-pSiNPs(L)) showed a consistent pattern in both assays (Figure 5C) where the smaller nanoparticle size exhibited slightly higher BBB penetration than their larger counterparts. The reason why we did not observe more significant differences between these two pSiNP sizes in the chips, may be the higher cytoadhesion of larger nanoparticles under flow conditions [31,57]. Larger nanoparticles tend to adhere and marginate closer to the channel wall than smaller nanoparticles due to their larger contact area [31,58,59]. However, it must be noted that size also affects biodistribution. For example, larger nanoparticles are more likely to be cleared by the spleen and liver, a factor that is currently not replicable in a dynamic BBB-on-a-chip model [60]. In addition, in vitro models lack other intricate complexities present in in vivo situations, such as the mononuclear phagocyte system and the wider immune system. On the other hand, ex vivo models allow a personalized medicine approach, allowing the study of cells from human pathologies. In general, ex vivo and humanized in vitro models hold superiority over rodent models [61].

In summary, the BBB-on-a-chip model revealed notable differences in the BBB crossing efficacy between pSiNPs with varying transferrin surface densities. Specifically, in this assay, pSiNPs with intermediate transferrin density exhibited the highest penetration efficacy, suggesting an optimal transferrin level for maximizing the BBB penetration performance for pSiNPs. The BBB results of the two sizes of pSiNPs are in agreement with the Transwell, indicating that smaller pSiNPs had a better BBB transportation.

## 4. Conclusions

PSiNPs were synthesized with two different sizes (average size of 203 and 420 nm) and decorated with different densities of transferrin by adjusting the ratio of bifunctional PEG linkers. We evaluated the effect of transferrin density and size on the hCMEC/D3 cell association and BBB transport of pSiNPs in both static and dynamic models. The results revealed that the surface density of transferrin on pSiNPs affects their hCMEC/D3 cell association, where increasing transferrin content from 0 nmol/mg to 3.8 nmol/mg enhances hCMEC/D3 association. The surface density of transferrin also affects the BBB transport of pSiNPs in the static Transwell assay. Here, the intermediate transferrin surface density showed the highest BBB transport. The use of a dynamic BBB-on-a-chip model resulted in more pronounced differences between the various surface ligand densities, where the intermediate transferrin surface density again showed the highest level of BBB crossing. These findings indicate that there is an optimal transferrin ligand density to maximize pSiNP delivery across the BBB, which could be influenced by their affinity to the transferrin receptor and underscore the role of flow in nanoparticle assessment. Furthermore, the smaller pSiNPs showed a consistently higher BBB penetration potential than the larger pSiNPs in all tested models, indicating that the size of pSiNPs significantly affects their BBB performance. Overall, our study demonstrated that surface density and size are important parameters in designing pSiNPs for crossing the BBB. Further in vivo experiments may provide additional validation of the BBB-on-a-chip results. In addition, the designed Tf-coated pSiNPs may have potential applications for the improved delivery of hydrophobic anti-cancer drugs such as doxorubicin and camptothecin to brain cancers like glioblastoma multiforme.

## Data Availability

Data are contained within the paper or supplementary information.

## Supplementary Materials

The following supporting information can be downloaded at: https://www.mdpi.com/article/10.3390/pharmaceutics15092271/s1, Figure S1: ATR-FTIR spectra of THC-pSiNPs and the subsequent hydrosilylation with undecylenic acid (UDA-pSiNPs). Figure S2: Characterization of THC-pSiNPs(S), UDA-pSiNPs(S), THC-pSiNPs(L) and UDA-pSiNPs(L). Figure S3: ATR-FTIR spectra of mPEG-NH_2_ and NH_2_-PEG-COOH in comparison with UDA-pSiNPs(S) and Tf-PEG/mPEG(1:50)-pSiNPs(S). Figure S4: BFTEM images Tf-PEG-pSiNPs(S) and Tf-PEG-pSiNPs(L) and their length (x-y dimension), thickness (z-dimension), and pore size. Figure S5: Cell viability results for hCMEC/D3 cells treated with different concentrations (5, 10, 25, 50 µg/mL) of modified pSiNPs for 48 h. Figure S6: Cy5 intensity of all modified pSiNPs on a per mass basis. Figure S7: Cellular uptake of Tf-PEG-pSiNPs(S) in hCMEC/D3 with z-stack scan. Figure S8: Validation of BBB-on-a-chip model. Figure S9: Transportation of Tf-pSiNPs from blood to brain channels after flowing for 4 h.
