# Supplementary material for: Influence of Surface Ligand Density and Particle Size on the Penetration of the Blood–Brain Barrier by Porous Silicon Nanoparticles
Source: Pharmaceutics. 2023 Sep 3;15(9):2271. doi: 10.3390/pharmaceutics15092271 (PMC10534822; doi:10.3390/pharmaceutics15092271)
Supplement: Supplementary file 1 [file pharmaceutics-15-02271-s001.zip › pharmaceutics-2496427-supplementary.pdf]

pharmaceutics

MDPI

Supplementary Materials

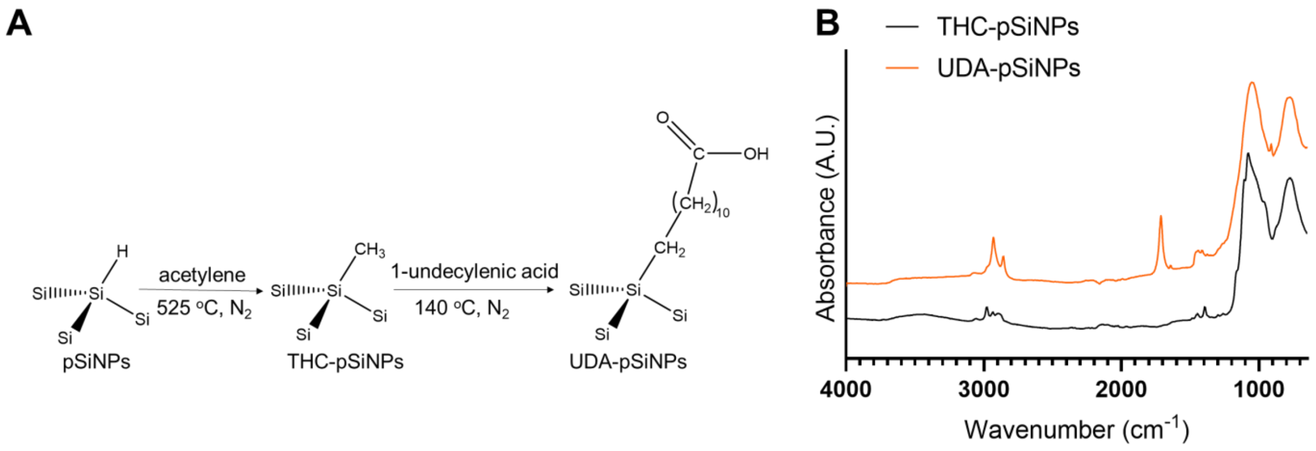

Figure S1. (A) Schematic synthesis of fabrication of UDA-pSiNPs from pSiNPs via acetylene and 1-undecylenic acid (B) ATR-FTIR spectra of THC-pSiNPs and the subsequent hydrosilylation with undecylenic acid (UDA-pSiNPs).

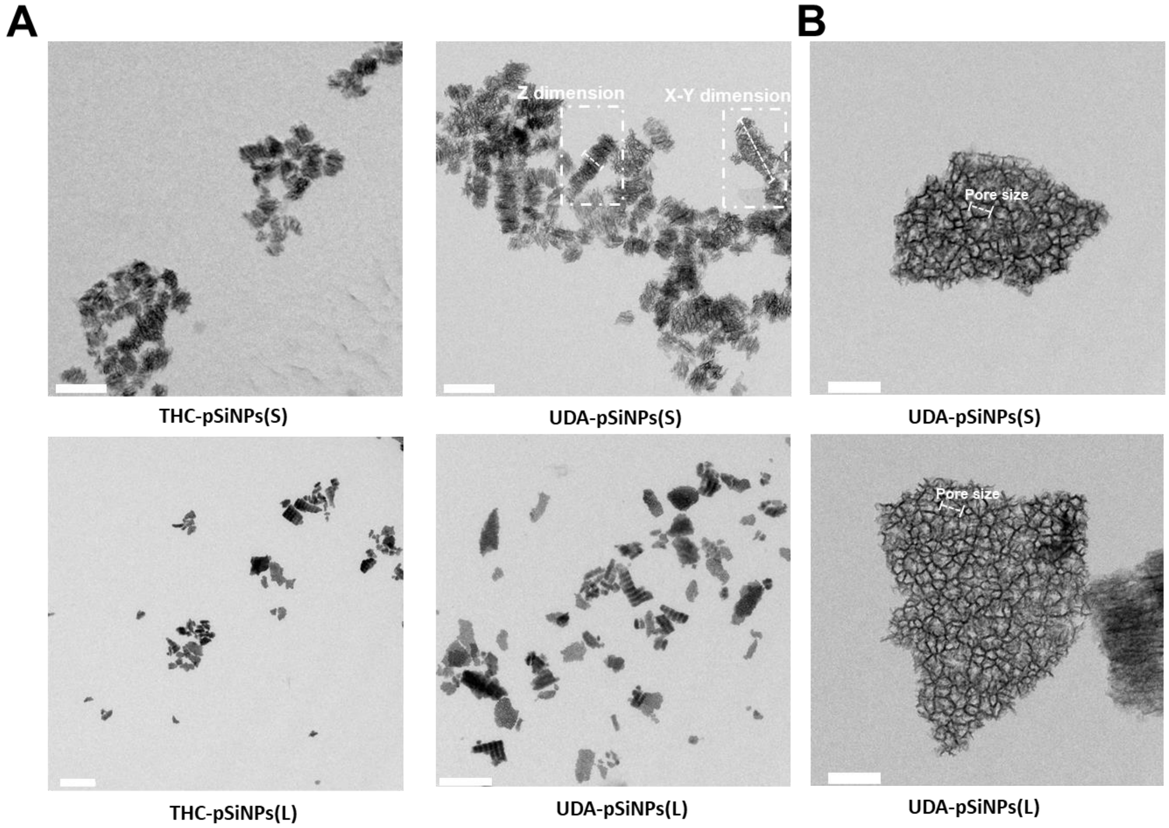

C

Sample	X-Y dimension (nm)	Z dimension (nm)	Pore size (nm)
THC-pSiNPs(S)	199.8 ± 40.3	86.3 ± 12.6	21.8 ± 5.3
UDA-pSiNPs(S)	188.1 ± 41.8	85.5 ± 13.8	22.0 ± 8.3
THC-pSiNPs(L)	427.4 ± 144.2	177.6 ± 83.2	26.8 ± 7.5
UDA-pSiNPs(L)	420.1 ± 151.8	161.9 ± 97.5	26.0 ± 6.3

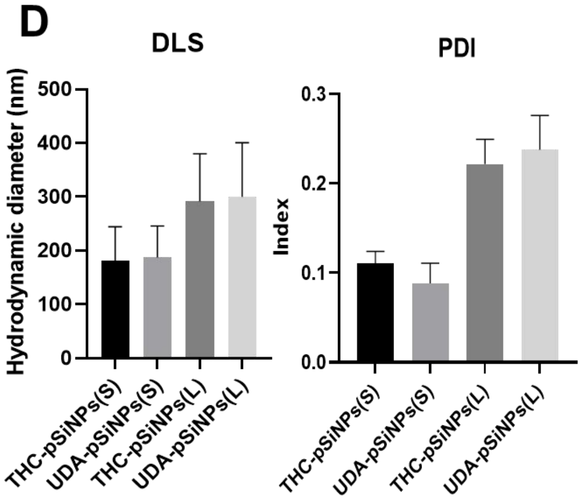

Figure S2. Characterization of THC-pSiNPs(S), UDA-pSiNPs(S), THC-pSiNPs(L) and UDA-pSiNPs(L). (A) Bright field transmission electron microscopy (BFTEM) images THC-pSiNPs(S), UDA-pSiNPs(S), THC-pSiNPs(L) and UDA-pSiNPs(L) in low magnification (Scale bar = 200 nm for small pSiNPs image and scale bar = 1000 nm for large pSiNPs image). (B) BFTEM images UDA-pSiNPs(S) and UDA-pSiNPs(L) in high magnification (Scale bar = 100 nm for both pSiNPs image). (C) Analysis of nanoparticles in their sizes in length (X-Y) and thickness (Z), and their pore size (the values are shown by mean ± sd, N=20) (D) The hydrodynamic diameter and polydispersity (PDI) of THC-pSiNPs(S), UDA-pSiNPs(S), THC-pSiNPs(L) and UDA-pSiNPs(L). Data are shown as mean ± sd, N = 3.

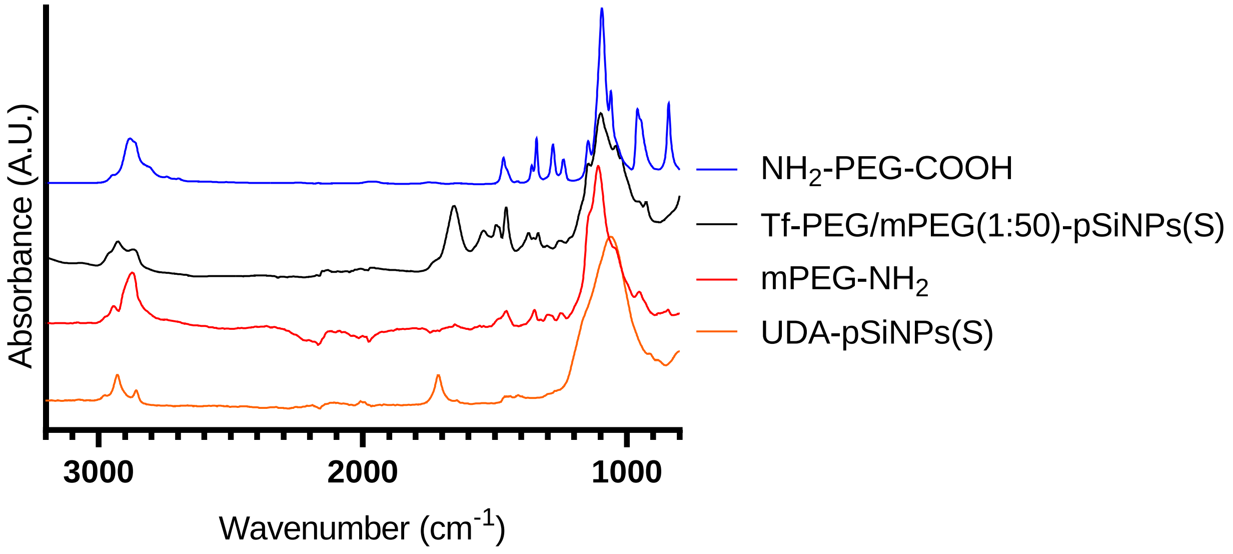

Figure S3. ATR-FTIR spectra of mPEG-NH_2 and NH_2-PEG-COOH in comparison with UDA-pSiNPs(S) and Tf-PEG/mPEG(1:50)-pSiNPs(S).

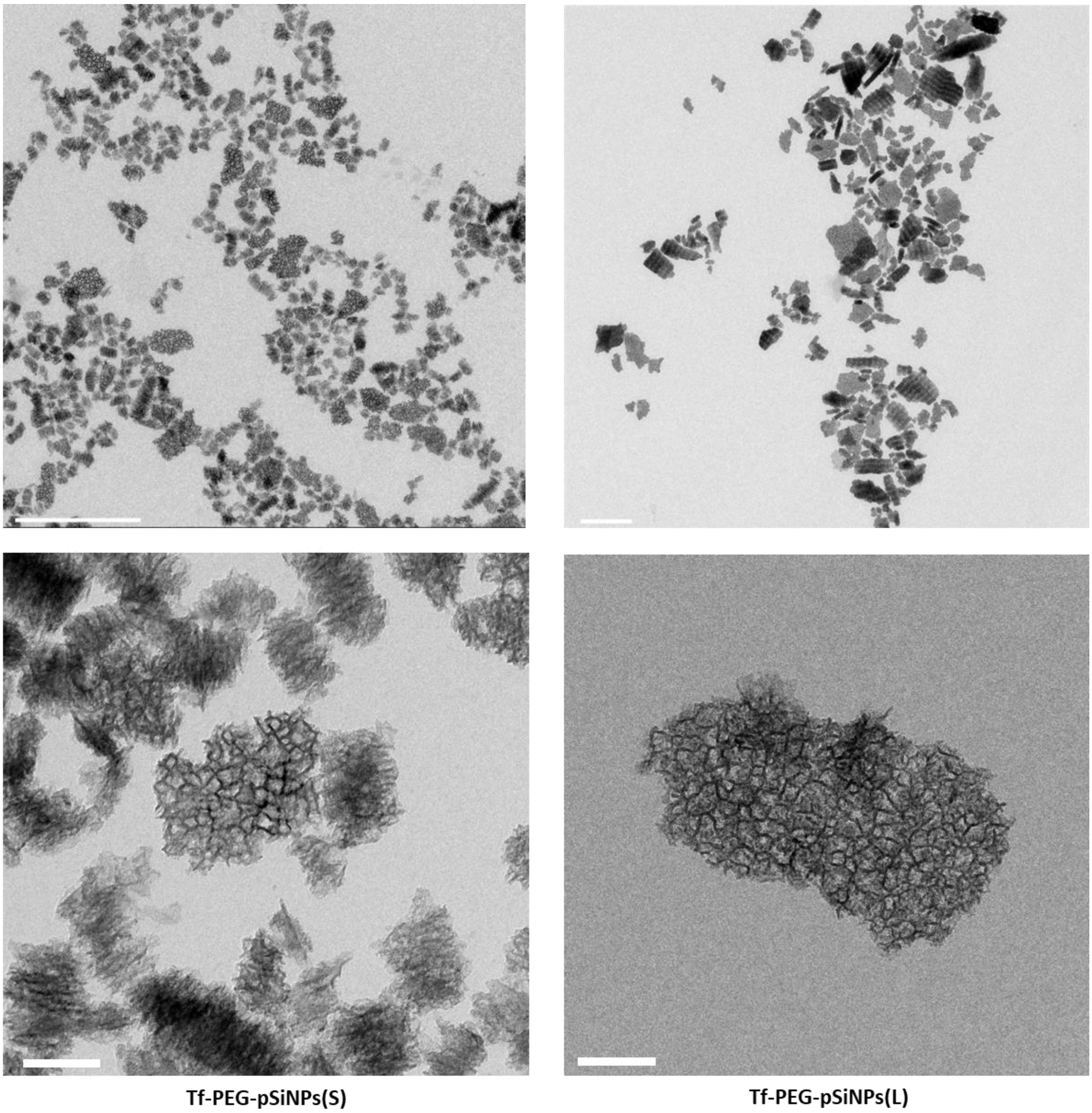

Sample	X-Y dimension (nm)	Z dimension (nm)	Pore size (nm)
Tf-PEG-pSiNPs(S)	203.5 ± 41.6	86.4 ± 14.2	25.7 ± 7.3
Tf-PEG-pSiNPs(L)	424.3 ± 131.4	172.2 ± 117.5	24.1 ± 4.5

Figure S4. BFTEM images Tf-PEG-pSiNPs(S) and Tf-PEG-pSiNPs(L) in low(top) and high (bottom) magnification and their length (x-y dimension), thickness (z-dimension), and pore size (values are shown by mean ± sd, N=80, Scale bar = 1000 nm in low magnification images, Scale bar = 100 nm in high magnification images).

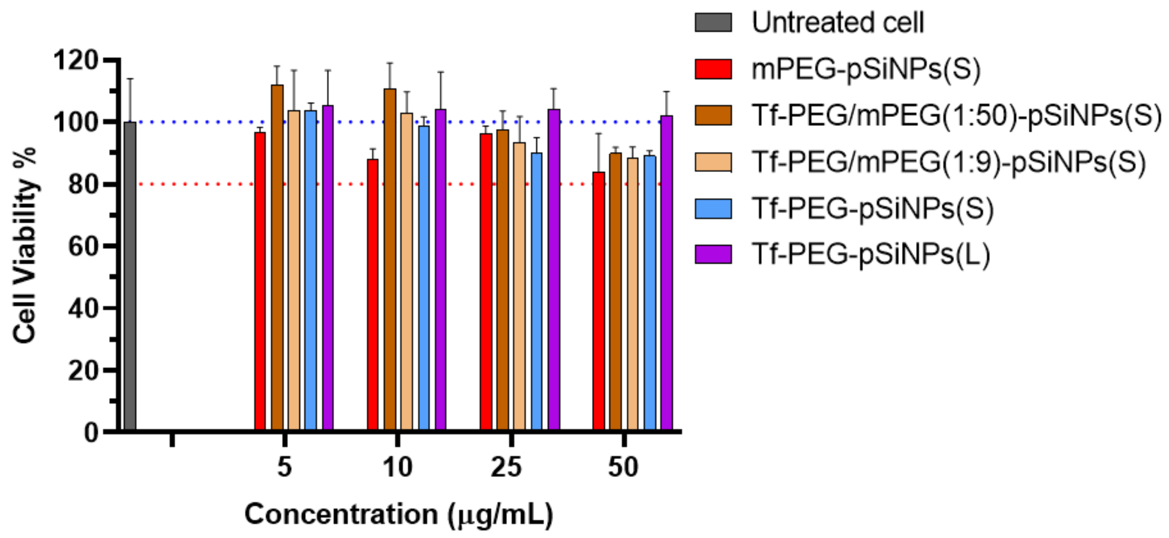

Figure S5. Cell viability results for hCMEC/D3 cells treated with different concentrations (5, 10, 25, 50 μg/mL) of modified pSiNPs for 48 h. Control (untreated hCMEC/D3 cells) was included. The values are shown by mean ± sd, N = 3.

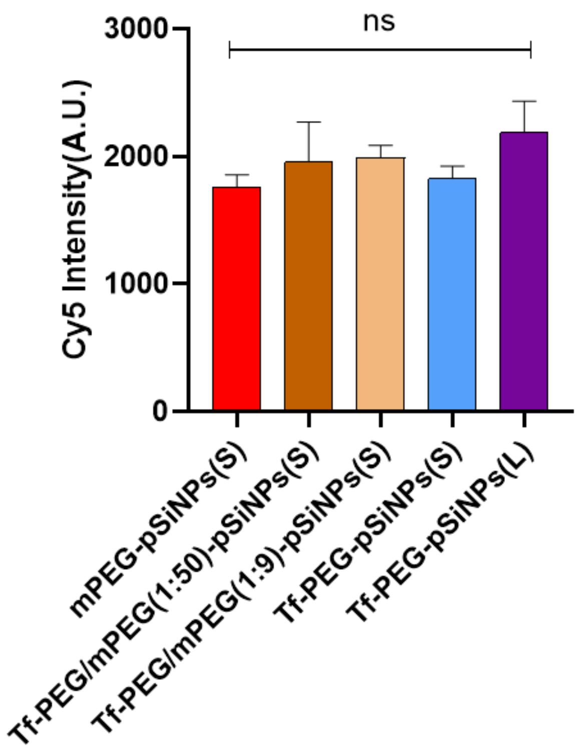

Figure S6. Cy5 intensity of all modified pSiNPs on a per mass basis. All samples maintained similar Cy5 intensity without significant differences. The values are shown by mean ± sd, N=3, One-way ANOVA test.

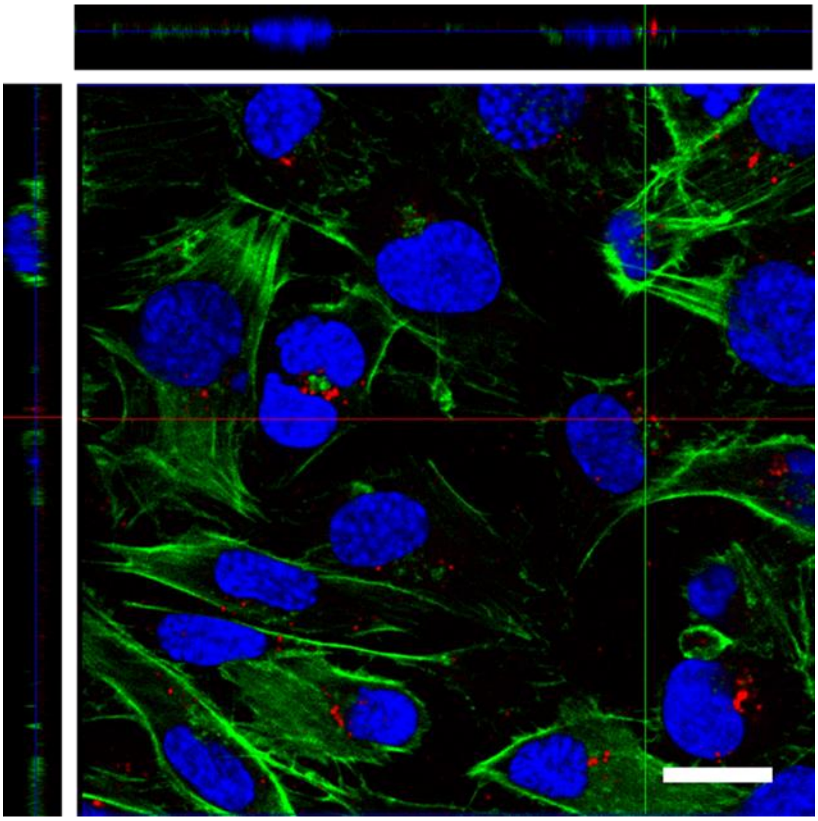

Figure S7. Cellular uptake of Tf-PEG-pSiNPs(S) in hCMEC/D3 with z-stack scan. (blue = nucleus, green = F-actin, red = nanoparticles, scale bar = 20 μm). The nanoparticle concentration was 5 μg/mL.

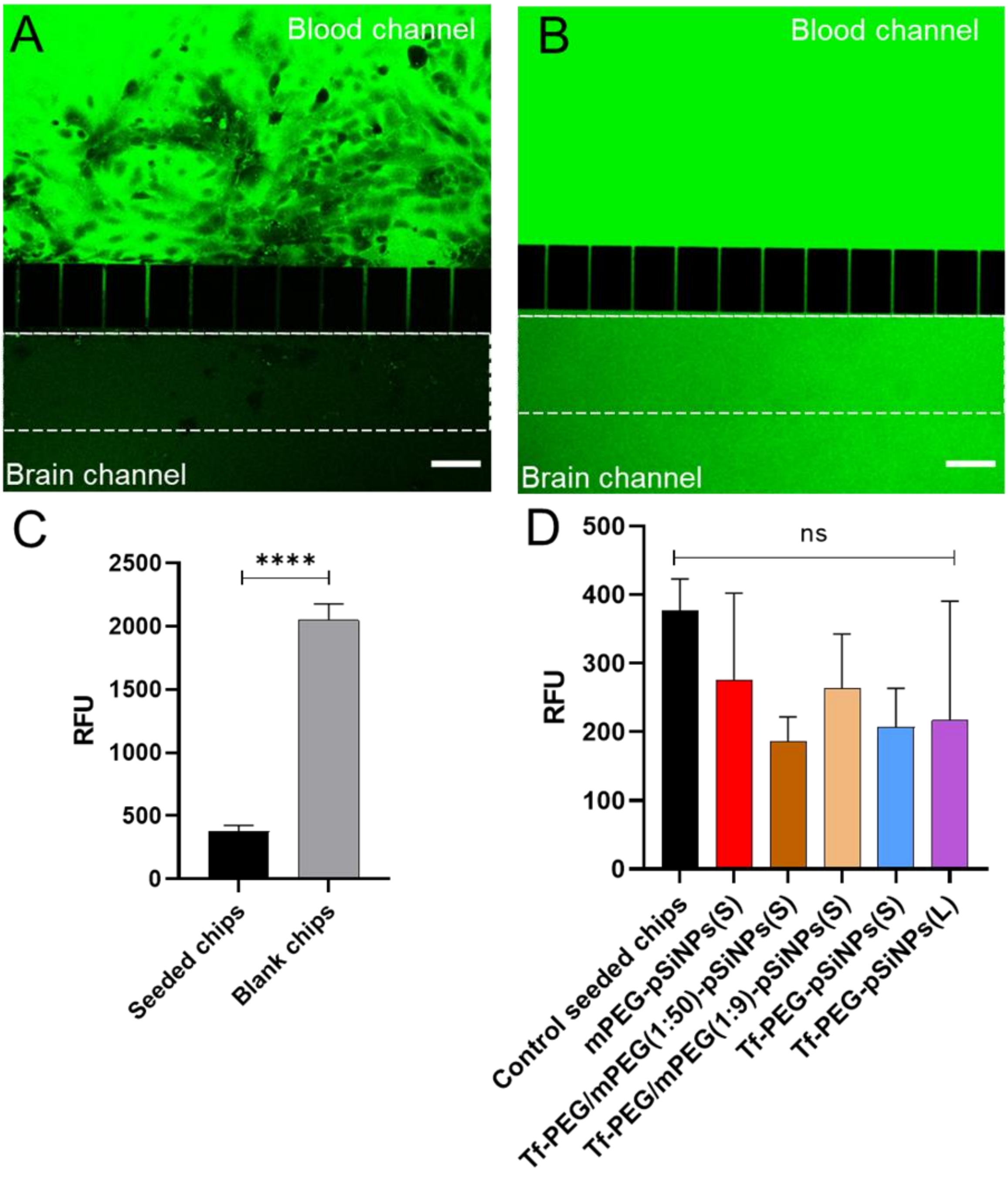

Figure S8. Validation of BBB-on-a-chip model. Comparison of FITC-dextran (10 kDa, 25 μg/mL) permeation into brain channels in (A) hCMEC/D3 seeded chips and (B) in blank control chips; scale bar: 50 μm. FITC-dextran largely remained in the blood channel in cultured chips while it was freely distributed in both channels in blank control chips. (C) Corresponding relative fluorescence intensity (RFU) of FITC-dextran in brain channels in cultured and control chips. RFU of brain channels in cultured chips was significantly lower than in control chips. The values are shown by mean ± sd, N = 3, Student's t-test, P**** < 0.0001. (D) RFU of FITC-dextran in brain channel in all tested NPs cultured chips showed no significant difference to control seeded chips. The values are shown by mean ± sd, N > 3, One-way ANOVA test.

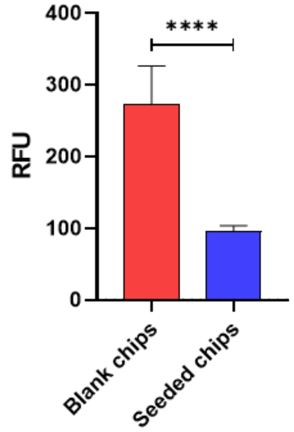

Figure S9. Transportation of Tf-pSiNPs from blood to brain channels after flowing for 4 h. Corresponding RFU of pSiNPs crossing blood channels in blank chips and hCMEC/D3 seeded chips. The values are shown by mean ± sd, N = 3, Student's t-test, $P^{****} < 0.0001$.
